# The Laborer Is Worthy of His Hire

**Published:** 1875-07

**Authors:** 


The Laborer is Worthy of his Hire.—Your committee would be remiss in duty not to have invited your serious attention to the general and lamentable inability of physicians to collect their fees, especially in our sparsely settled rural districts. That reform is imperatively demanded in this matter of life-blood—of just remuneration for professional services—is so evident to all that to state the fact is but to prove it. "*We cannot collect our bills*" is more than ever the universal, depressing and almost despairing cry of country physicians. This complaint not only comes from the "hard times," now so pinching to all classes and occupations, but it is based mainly upon the *depravity of the times*, as connected with the proverbial ingratitude of the public to physicians, and the well-known disposition of a very large number of patients not to pay the doctor. This inability, since the war, of physicians to collect their bills does not originate from financial inability of our patients to pay, poor though so many of them are, but it arises from the prevailing demoralization in regard to obligations to pay just debts, and to the advantages which so many mean men take of the shield of the homestead exemption law. Herein exists additional demand for the union of the profession everywhere, in order that, while doing justice to our patients, we may receive justice, and ourselves and families be saved from extreme want and poverty. The operation of causes thus alluded to has entailed and is entailing upon the profession an overwhelming load of poverty, with its attendant anxiety and distress.

The picture presented is correct, except that it is not full enough. The sad condition on these vital points presents an inexorable demand for improvement and reform. With the broad fact staring us in the face that swindlers and dishonest debtors pray and impose upon our liberal and humane profession far more than upon any other class or calling, it is our high duty to appeal to our brethren in every section of North Carolina to arouse from their lethargy, and meet the emergencies now upon us. To hesitate is to doom the profession to a still sadder fate, and to drive a yet larger number of our brightest minds and noblest members to other pursuits for a living.—*Eastern Medical Asso., N. C.*

